# Changes in sleep disturbances and quality of life associated with Istradefylline in patients with Parkinson's disease: an exploratory polysomnographic study

**DOI:** 10.1016/j.prdoa.2026.100512

**Published:** 2026-10-13

**Authors:** Noriyuki Miyaue, Hayato Yabe

**Affiliations:** a Department of Clinical Pharmacology and Therapeutics, Ehime University Graduate School of Medicine, Toon, Ehime, Japan; b Department of Neurology, Saiseikai Matsuyama Hospital, Matsuyama, Ehime, Japan

**Keywords:** Parkinson's disease, Istradefylline, Sleep disturbance, Polysomnography, REM sleep, Quality of life

## Abstract

**Background:**

Sleep disturbances are common non-motor symptoms in Parkinson's disease (PD) and substantially impair quality of life (QOL). Although istradefylline, an adenosine A2A receptor antagonist, is widely used to improve motor fluctuations, its effects on subjective sleep complaints and objective sleep architecture remain unclear.

**Methods:**

In this exploratory, single-center, open-label study, we evaluated changes in subjective sleep quality and polysomnographic findings following 12 weeks of istradefylline treatment in patients with PD. Nine patients underwent clinical assessments and overnight polysomnography at baseline and 12 weeks after initiating istradefylline (20 mg/day). Sleep quality, QOL, fatigue, and motor symptoms were assessed using validated clinical scales.

**Results:**

Following istradefylline initiation, improvements were observed in Parkinson's Disease Sleep Scale-2 (PDSS-2), Parkinson's Disease Questionnaire-39 summary index (PDQ-39 SI), Parkinson Fatigue Scale, and Movement Disorder Society-Unified Parkinson's Disease Rating Scale (MDS-UPDRS) Part IV scores. Changes in PDSS-2 scores were significantly correlated with changes in PDQ-39 SI (ρ = 0.76, *p* = 0.017), although both measures were subjective and obtained at the same assessment. Regarding objective sleep measures, most polysomnographic parameters did not significantly change; rapid eye movement (REM) sleep percentage increased numerically, but the change was not statistically significant (*p* = 0.058).

**Conclusions:**

In this small uncontrolled study, initiation of istradefylline was associated with improvements in subjective sleep quality, fatigue, and QOL, whereas objective polysomnographic parameters showed limited changes. These findings are exploratory and hypothesis-generating and require confirmation in larger controlled studies.

## Highlights

• Istradefylline improved subjective sleep, fatigue, and QOL in PD.
• Changes in subjective sleep quality were strongly correlated with changes in QOL.
• Full overnight PSG showed a numerical increase in REM sleep percentage.
• Subjective improvements occurred without significant changes in PSG parameters.

## 1 Introduction

Parkinson's disease (PD) is a progressive neurodegenerative disorder characterized not only by motor symptoms but also by a broad spectrum of non-motor symptoms [1]. Sleep disturbances are among the most prevalent and burdensome non-motor symptoms in patients with PD and substantially impair quality of life (QOL) [2]. Moreover, sleep dysfunction has been shown to affect QOL across different stages of disease progression [3]. Sleep-related problems in PD are multifactorial and include nocturnal motor symptoms, insomnia, fragmented sleep, excessive daytime sleepiness, and rapid eye movement (REM) sleep behavior disorder [4]. Polysomnographic studies have demonstrated abnormalities in sleep architecture in PD, including reduced REM sleep and impaired sleep efficiency [5]. In addition, fatigue is a common and disabling non-motor symptom and is closely associated with sleep dysfunction and reduced QOL [6].

Istradefylline is a selective adenosine A2A receptor antagonist approved as an adjunctive treatment for levodopa-treated patients with PD experiencing wearing-off phenomena. Adenosine A2A receptors are highly expressed in the striatum, where they modulate the indirect pathway of the basal ganglia. Interestingly, adenosine also plays a crucial role in the regulation of the sleep-wake cycle, and A2A receptors are involved in the sleep-promoting effects of adenosine [7]. While several clinical studies have suggested that istradefylline may be associated with improvements in subjective non-motor symptoms, such as daytime sleepiness and fatigue [8], its relationship with objective sleep architecture measured by polysomnography (PSG) remains poorly understood. In addition, improvement in nocturnal motor symptoms or motor fluctuations could indirectly influence perceived sleep quality, providing another potential mechanism by which istradefylline may affect sleep-related symptoms.

Given the limited evidence regarding the association of istradefylline initiation with subjective and objective sleep parameters in PD, further investigation is warranted. Therefore, this study aimed to evaluate changes in objective sleep parameters using full overnight PSG and subjective clinical scales, including QOL, sleep quality, and fatigue, over 12 weeks following initiation of istradefylline in patients with PD.

## 2 Methods

### 2.1 Study design and participants

This single-center, open-label study was conducted at Saiseikai Matsuyama Hospital between October 2018 and March 2025. We enrolled patients with PD who met the Movement Disorder Society clinical diagnostic criteria [9]. Patients were eligible if they were scheduled to initiate istradefylline as part of routine clinical care and provided written informed consent to participate in the present study. No sleep-specific inclusion criterion was applied. Clinical assessments were performed at baseline and 12 weeks after initiation of istradefylline (20 mg/day). Istradefylline was administered once daily after breakfast throughout the 12-week period. Exclusion criteria were as follows: (1) a history of istradefylline treatment, and (2) any adjustments to the antiparkinsonian medication regimen within one month prior to enrollment.

This study was designed as an exploratory pilot study; no formal a priori sample size calculation was performed. This study protocol was approved by the Institutional Review Board for Clinical Research Ethics of Saiseikai Matsuyama Hospital and conducted in accordance with the Declaration of Helsinki. Written informed consent was obtained from all participants prior to enrollment.

### 2.2 Clinical assessments

Clinical evaluations were performed at baseline and during follow-up. Motor and non-motor symptoms were evaluated during the ON-medication state using the Movement Disorder Society-Unified Parkinson's Disease Rating Scale (MDS-UPDRS) [10]. QOL was assessed using the Parkinson's Disease Questionnaire-39 summary index (PDQ-39 SI) [11]. Individual PDQ-39 domain scores were also evaluated as exploratory outcomes. Sleep-related symptoms were assessed using the Parkinson's Disease Sleep Scale-2 (PDSS-2) [12], including its component domains, and excessive daytime sleepiness was evaluated using the Japanese version of the Epworth Sleepiness Scale (ESS) [13]. Fatigue was evaluated using the Parkinson Fatigue Scale (PFS) [14]. Demographic and clinical data, including age, sex, disease duration, Hoehn–Yahr stage, cognitive status assessed using the Mini-Mental State Examination (MMSE) and Japanese version of the Montreal Cognitive Assessment (MoCA-J), and concomitant medications, were collected at baseline. Levodopa-equivalent daily dose (LEDD) was calculated according to a standard conversion formula [15].

### 2.3 Polysomnography

Full overnight PSG was performed using a digital polygraph system (Alice PDx; Philips Respironics, Murrysville, PA, USA) at baseline and 12 weeks post-treatment. All PSG data were transferred to the Philips Scoring Center (Tokyo, Japan) for centralized analysis. PSG recordings were scored by experienced registered sleep technologists who were blinded to the treatment time point. Sleep stages and respiratory events were scored according to the criteria established by the American Academy of Sleep Medicine Manual. An adaptation night was not performed. The PSG recordings were conducted under the same clinical protocol at baseline and week 12. Istradefylline was administered once daily after breakfast throughout the 12-week treatment period. Inter-rater and intra-rater reliability were not formally assessed. REM atonia and RBD status were not systematically assessed in the present study.

### 2.4 Statistical analysis

Continuous variables are presented as median [interquartile range (IQR)]. Intragroup comparisons between baseline and 12 weeks were performed using the Wilcoxon signed-rank test. Effect sizes were calculated as the absolute value of the standardized Wilcoxon signed-rank statistic divided by the square root of the number of non-zero paired differences (*r* = |Z|/√N), where N represents the number of non-zero paired differences included in the analysis. Correlations between changes in clinical variables were assessed using Spearman's rank correlation coefficient. Because this was an exploratory pilot study, multiple clinical and PSG outcomes were examined without formal adjustment for multiple comparisons. Accordingly, *p*-values were interpreted as exploratory rather than confirmatory. A two-sided *p*-value <0.05 was considered statistically significant. Confidence intervals for the Spearman correlation coefficient were calculated using a bias-corrected and accelerated bootstrap approach. All statistical analyses were conducted using R software (version 4.5.2: R Foundation for Statistical Computing, Vienna, Austria).

## 3 Results

### 3.1 Patient characteristics

Ten patients were initially enrolled; however, one patient was excluded from the follow-up evaluation due to concerns regarding coronavirus disease 2019 infection after completing only the baseline assessment. Consequently, 9 patients (7 males, 2 females) were included in the final analysis. The median age was 71 [66–79] years; the median disease duration was 6 [5], [6], [7], [8] years, and the median LEDD was 700 [679–882] mg/day. Individual clinical characteristics, cognitive assessments, Hoehn–Yahr stage, and concomitant medications are presented in Supplementary Table S1. No changes in antiparkinsonian medications or LEDD occurred during the 12-week treatment period, and Hoehn–Yahr stage remained unchanged. Two patients were receiving sleep-related medications at baseline: clonazepam in one patient and suvorexant in another. Neither medication nor its dosage was changed during the 12-week study period. None of the patients routinely used caffeine or alcohol.

### 3.2 Clinical outcomes

Changes in clinical outcomes after 12 weeks are shown in Table 1 and Fig. 1. The PDQ-39 SI significantly decreased from a median of 41.5 [29.1–47.5] at baseline to 23.2 [9.9–38.2] at week 12 (*p* = 0.009, *r* = 0.87). Regarding sleep-related outcomes, the total PDSS-2 scores significantly improved from 26 [22–42] to 20 [11–29] (*p* = 0.033, *r* = 0.71). Among the PDSS-2 domains, PD-related symptoms at night and disturbed sleep also showed significant reductions (Supplementary Table S2). ESS scores did not significantly change after treatment (11 [11], [12], [13], [14], [15] vs. 11 [8], [9], [10], [11], [12], [13], [14], [15], [16], [17], *p* = 0.260, *r* = 0.38). PFS scores decreased from 62 [58–68] to 43 [38–55] (*p* = 0.024, *r* = 0.75). Regarding motor complications, the MDS-UPDRS Part IV score showed a significant decrease from 4 [3], [4], [5] to 2 [0–5] (*p* = 0.034, *r* = 0.71). MDS-UPDRS Part I and total scores did not reach statistical significance (*p* = 0.074 and *p* = 0.097, respectively), and no significant changes were observed in Parts II or III. Exploratory analyses of the individual PDQ-39 domains are presented in Supplementary Table S3.

**Table 1:** Clinical and polysomnographic outcomes at baseline and week 12.

	Baseline	Week 12	*p* value	Effect size (*r*)
**Quality of life**				
PDQ-39 SI	41.5 [29.1–47.5]	23.2 [9.9–38.2]	**0.009**	0.87
**Sleep-related scales**				
PDSS-2 score	26 [22–42]	20 [11–29]	**0.033**	0.71
ESS score	11 [11–15]	11 [8–17]	0.260	0.38
**Fatigue scale**				
PFS score	62 [58–68]	43 [38–55]	**0.024**	0.75
**MDS-UPDRS**				
Part I	15 [11−20]	12 [8–17]	0.074	0.60
Part II	21 [15–27]	16 [9–25]	0.306	0.34
Part III	39 [35–48]	39 [31–45]	0.342	0.32
Part IV	4 [3–5]	2 [0–5]	**0.034**	0.71
Total score	85 [70–85]	69 [53–75]	0.097	0.55
**Polysomnographic parameters**				
Total sleep time (min)	295 [229–375.5]	279 [258–383.5]	0.407	0.28
Wake after sleep onset (min)	247.5 [194.5–329.5]	302.5 [127–346.5]	0.477	0.24
Sleep efficiency (%)	54.4 [41–64.8]	46 [43.8–71.7]	0.529	0.21
REM sleep (%)	1.5 [0–13.4]	4.7 [2.9–15.9]	0.058	0.63
N1 sleep (%)	16.9 [14.9–29.3]	23.3 [22.2–25.2]	0.554	0.20
N2 sleep (%)	61.5 [53.6–70.7]	66.8 [61.3–72.3]	1.000	0.00
N3 sleep (%)	0 [0–0]	0 [0–3.6]	0.855	0.00

Data are presented as median [interquartile range]. Nominal *p* values (< 0.05) are shown in bold. Comparisons between baseline and week 12 were performed using the Wilcoxon signed-rank test. Effect sizes were calculated as the absolute value of the standardized Wilcoxon signed-rank statistic divided by the square root of the number of non-zero paired differences (*r* = |Z|/√N), where N represents the number of non-zero paired differences included in the analysis. No adjustment for multiple comparisons was applied because of the exploratory nature of this pilot study.

PDQ-39 SI, Parkinson's Disease Questionnaire-39 summary index; PDSS-2, Parkinson's Disease Sleep Scale-2; ESS, Epworth Sleepiness Scale; PFS, Parkinson Fatigue Scale; MDS-UPDRS, Movement Disorder Society-sponsored revision of the Unified Parkinson's Disease Rating Scale; REM, rapid eye movement.

**Fig. 1:**
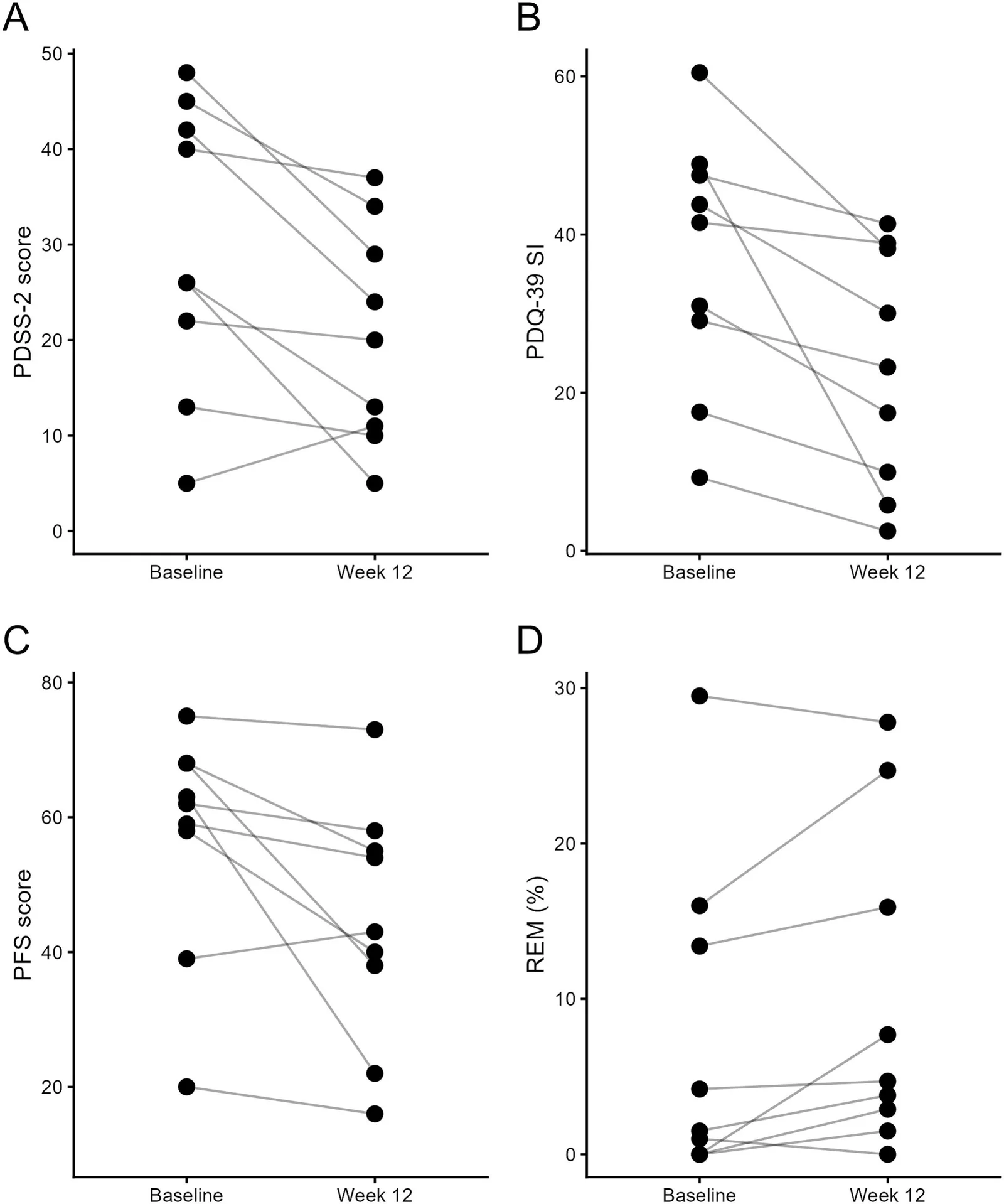
Changes in clinical and polysomnographic outcomes from baseline to week 12. Individual patient trajectories are shown for (A) PDSS-2 score, (B) PDQ-39 SI, (C) PFS score, and (D) REM sleep percentage. Each line represents an individual patient. PDSS-2, Parkinson's Disease Sleep Scale-2; PDQ-39 SI, Parkinson's Disease Questionnaire-39 summary index; PFS, Parkinson Fatigue Scale; REM, rapid eye movement.

### 3.3 Polysomnographic findings

Objective sleep measures obtained via PSG did not show statistically significant changes after treatment (Table 1 and Supplementary Table S4). Total sleep time, wake after sleep onset, and sleep efficiency did not significantly change. REM sleep percentage increased numerically from 1.5% [0–13.4] to 4.7% [2.9–15.9], but this change did not reach statistical significance (*p* = 0.058, *r* = 0.63) (Fig. 1D). Proportions of N1, N2, and N3 sleep stages also did not significantly change. Additional PSG parameters, including REM latency, arousal index, apnea-hypopnea index (AHI), oxygen desaturation index (ODI), periodic limb movements during sleep index (PLMS index), and periodic limb movements during sleep arousal index (PLMS-arousal index), showed no statistically significant changes (Supplementary Table S4).

### 3.4 Correlation and clinical changes

Correlation analysis revealed that changes in PDSS-2 scores were significantly and positively correlated with changes in PDQ-39 SI (Spearman's ρ = 0.76, 95% CI −0.05–0.98; *p* = 0.017), suggesting that subjective sleep improvement was associated with enhanced QOL (Fig. 2). No significant correlations were found between changes in PDSS-2 scores and changes in PFS scores, REM sleep percentage, or MDS-UPDRS total scores.

**Fig. 2:**
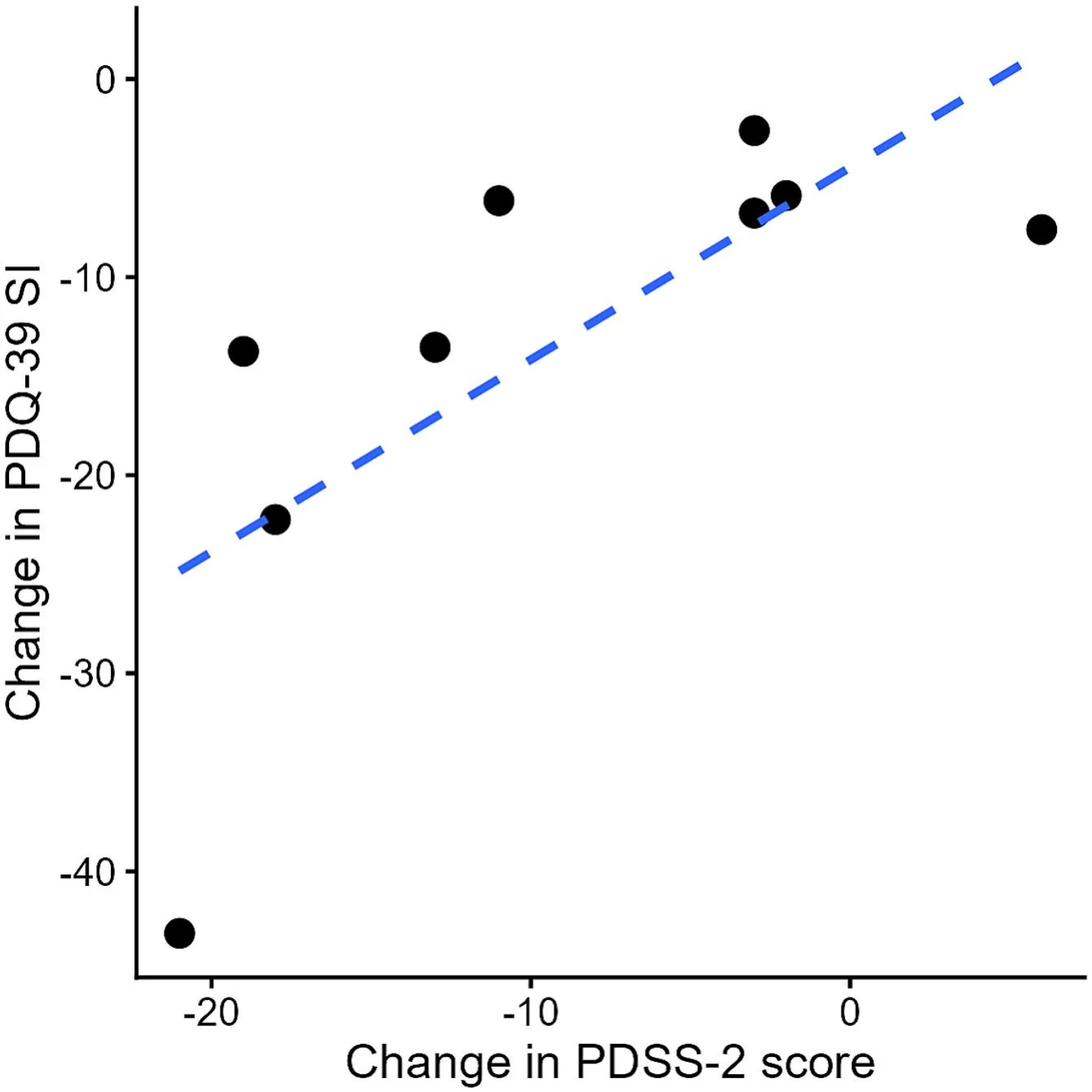
Correlation between changes in PDSS-2 score and PDQ-39 summary index. Scatter plot showing the relationship between changes from baseline to week 12. A significant positive correlation was observed between changes in PDSS-2 score and PDQ-39 SI (Spearman's ρ = 0.76, *p* = 0.017). PDSS-2, Parkinson's Disease Sleep Scale-2; PDQ-39 SI, Parkinson's Disease Questionnaire-39 summary index.

## 4 Discussion

In this exploratory, single-center, open-label study, initiation of istradefylline was associated with improvements in subjective sleep symptoms, QOL, and fatigue over 12 weeks in patients with PD. The PDSS-2 and PDQ-39 SI both improved, and changes in PDSS-2 were positively correlated with changes in PDQ-39 SI. In contrast, objective sleep parameters assessed by overnight PSG showed no statistically significant changes. Although REM sleep percentage increased numerically, the change did not reach statistical significance (*p* = 0.058). These findings should therefore be considered preliminary and hypothesis-generating rather than evidence of a causal treatment effect.

Sleep disturbances are highly prevalent in PD and are strongly associated with impaired QOL [2]. In this study, PDSS-2 scores decreased after initiation of istradefylline, particularly in domains related to nocturnal PD symptoms and disturbed sleep. At the same time, PDQ-39 SI also improved, and changes in PDSS-2 were positively correlated with changes in PDQ-39 SI. This association is clinically relevant because sleep disturbances may contribute to impaired daytime functioning and overall well-being in patients with PD. However, the correlation should be interpreted cautiously, given the small sample size and the wide 95% CI. Both PDSS-2 and PDQ-39 are self-reported measures obtained at the same assessment, and their constructs may partially overlap. Furthermore, the uncontrolled design prevents determination of whether improvement in sleep symptoms directly resulted in improvement in QOL. The improvement in QOL may also have been influenced by changes in motor fluctuations. MDS-UPDRS Part IV scores decreased significantly during the study period, whereas the total MDS-UPDRS score did not reach statistical significance. Therefore, improvement in motor complications may have contributed to the observed changes in sleep-related symptoms and QOL.

Previous studies investigating the effects of istradefylline on sleep symptoms in PD have reported inconsistent findings. An open-label study demonstrated significant improvement in excessive daytime sleepiness assessed using the ESS, whereas total PDSS-2 scores did not significantly change after 3 months of treatment [16]. Similarly, another study reported improvement in daytime sleepiness and freezing of gait without worsening nocturnal disability [17]. In this study, PDSS-2 scores improved, whereas ESS scores did not significantly change. The improvement in PDSS-2 was mainly observed in domains related to nocturnal PD symptoms and disturbed sleep. These discrepancies may be attributable to differences in patient characteristics, baseline sleep disturbances, disease severity, concomitant medications, and study design. These findings therefore extend previous clinical observations by suggesting that subjective sleep-related symptoms may change after initiation of istradefylline even when daytime sleepiness measured by the ESS does not show a clear change. However, given the small sample size and uncontrolled design, this observation requires confirmation in larger prospective studies.

Despite improvement in subjective sleep-related symptoms, none of the assessed PSG parameters showed a statistically significant change after 12 weeks. REM sleep abnormalities are common in PD and may reflect neurodegeneration involving brainstem structures associated with sleep regulation [18], [19]. REM sleep percentage increased numerically from 1.5% to 4.7%, but this difference did not reach statistical significance (*p* = 0.058). Therefore, the present study does not provide evidence that istradefylline improves objective sleep architecture. The low baseline REM and N3 sleep percentages may reflect the substantial sleep disruption observed in this cohort, although the absence of an adaptation night and the small sample size make interpretation difficult. The discrepancy between subjective and objective sleep measures is noteworthy. Subjective sleep symptoms and conventional PSG parameters may capture different aspects of sleep disturbance in PD, and improvement in perceived sleep quality may not necessarily be accompanied by measurable changes in conventional sleep architecture [5].

The mechanisms underlying the association between istradefylline initiation and improvement in subjective sleep-related symptoms remain unclear. Although adenosine A2A receptor antagonists are generally associated with arousal-promoting effects, the present exploratory findings suggest that subjective sleep quality may improve after istradefylline treatment. Adenosine signaling plays an important role in sleep–wake regulation, and adenosine A2A receptor antagonism may modulate arousal systems and sleep regulation [20], [21]. In addition, clinical improvement in motor fluctuations may indirectly affect nocturnal symptoms and perceived sleep quality. The significant reduction in MDS-UPDRS Part IV scores in the present study is consistent with this possibility. However, the present study was not designed to distinguish direct effects of A2A receptor antagonism on sleep from indirect effects mediated through motor symptoms or other non-motor symptoms. The observed changes should therefore be interpreted as associations rather than evidence of a specific pharmacological mechanism.

This study has several limitations. First, the sample size was very small (*n* = 9), and no formal sample size calculation was performed because this was an exploratory pilot study. The study therefore had limited statistical power and limited generalizability. Second, the study used a single-center, open-label, uncontrolled before-after design. Consequently, placebo effects, expectancy effects, regression to the mean, spontaneous fluctuations in symptoms, and other time-dependent factors cannot be excluded. The study therefore cannot establish a causal relationship between istradefylline initiation and the observed clinical changes. Third, multiple clinical and PSG outcomes were examined without formal correction for multiple comparisons. Accordingly, the statistically significant findings should be interpreted as exploratory, and the possibility of false-positive findings cannot be excluded. Fourth, concomitant medications were not standardized. Although antiparkinsonian medications and LEDD remained unchanged during the study period, two participants were receiving sleep-related medications at baseline (clonazepam or suvorexant), and neither medication nor its dosage was changed before or during the study period. These medications may nevertheless have influenced sleep and PSG outcomes. Other antiparkinsonian medications also differed among participants, which may have influenced sleep and QOL outcomes. Fifth, PSG was performed without an adaptation night, and inter-rater and intra-rater reliability were not formally assessed. RBD status and REM atonia were also not systematically assessed. These factors may limit interpretation of the PSG findings. Finally, both PDSS-2 and PDQ-39 are subjective measures, and their simultaneous improvement and correlation may partly reflect overlapping constructs or common response tendencies. Although changes were observed in these measures, this small study was not designed to establish the minimal clinically important difference of these measures in the context of istradefylline treatment.

Nevertheless, this study has several strengths. First, subjective clinical assessments and objective overnight PSG were performed longitudinally in the same patients, allowing changes in perceived sleep symptoms to be compared with objective sleep parameters. Second, PSG assessment included multiple sleep architecture, respiratory, arousal, and periodic limb movement parameters. Third, the study was conducted under routine clinical conditions, providing preliminary real-world information regarding sleep-related changes following initiation of istradefylline.

Future studies should include larger, adequately powered cohorts and randomized controlled designs with appropriate comparator groups. Repeated PSG measurements under standardized conditions, including adaptation nights when feasible, would help determine whether changes in subjective sleep symptoms are accompanied by reproducible changes in sleep architecture. Future studies should also systematically assess RBD, REM atonia, sleep-disordered breathing, periodic limb movements, daytime sleepiness, motor fluctuations, and concomitant medications. It would also be important to determine whether changes in PDSS-2 and QOL are clinically meaningful and whether they are mediated primarily by improvements in nocturnal motor symptoms, daytime functioning, fatigue, or other mechanisms. Such studies may clarify whether the effects observed in the present pilot study represent a reproducible clinical signal or reflect nonspecific changes associated with treatment initiation.

## 5 Conclusions

In this small, uncontrolled exploratory study, initiation of istradefylline was associated with improvements in subjective sleep symptoms, fatigue, and QOL in patients with PD. Objective PSG parameters showed no statistically significant changes, and the numerical increase in REM sleep percentage did not reach statistical significance. These findings are preliminary and hypothesis-generating and do not establish a causal effect of istradefylline on sleep. Larger controlled studies with standardized PSG assessment are warranted to determine whether the observed subjective improvements are reproducible and whether they are accompanied by objective changes in sleep.

## CRediT authorship contribution statement

**Noriyuki Miyaue:** Writing – review & editing, Writing – original draft, Resources, Methodology, Investigation, Formal analysis, Data curation, Conceptualization. **Hayato Yabe:** Writing – review & editing, Resources, Data curation, Conceptualization.

## Compliance with ethical standards

This study protocol was approved by the Institutional Review Board for Clinical Research Ethics of Saiseikai Matsuyama Hospital and conducted in accordance with the Declaration of Helsinki. Written informed consent was obtained from all participants prior to enrollment.

## Ethics declaration

Written informed consent to take part in the study and to publish the article has been obtained from all participants or their legal representatives. The privacy rights of participants have been observed.

This study was performed in compliance with relevant laws, regulatory frameworks and guidelines where the research took place. This study was approved by the Institutional Review Board for Clinical Research Ethics of Saiseikai Matsuyama Hospital (Approval No. S18–028).

## Funding

This research did not receive any specific grant from funding agencies in the public, commercial, or not-for-profit sectors.

## Declaration of competing interest

The authors have no conflict of interest to report.

## Data Availability

The datasets generated and/or analyzed during the current study are available from the corresponding author upon reasonable request.
