# Supplementary material for: Changes in sleep disturbances and quality of life associated with Istradefylline in patients with Parkinson's disease: an exploratory polysomnographic study
Source: Clin Park Relat Disord. 2026 Oct 13;15:100512. doi: 10.1016/j.prdoa.2026.100512 (PMC13693060; doi:10.1016/j.prdoa.2026.100512)
Supplement: Supplementary file 1 [file mmc1.docx]

**Supplementary Table S1. Individual clinical characteristics and concomitant medications at baseline.**

|  | *Case1* | *Case2* | *Case3* | *Case4* | *Case5* | *Case6* | *Case7* | *Case8* | *Case9* |
| --- | --- | --- | --- | --- | --- | --- | --- | --- | --- |
| Age, years | 85 | 63 | 79 | 59 | 72 | 70 | 71 | 66 | 79 |
| Sex | Male | Female | Female | Male | Male | Male | Male | Male | Male |
| Disease duration, years | 6 | 8 | 3 | 10 | 4 | 6 | 5 | 7 | 9 |
| Hoehn–Yahr stage | 3 | 3 | 3 | 3 | 4 | 3 | 3 | 4 | 3 |
| MMSE | 28 | 29 | 28 | 29 | 21 | 30 | 22 | 30 | 26 |
| MoCA-J | 22 | 22 | 24 | 25 | 15 | 20 | 14 | 26 | 19 |
| LEDD, mg/day | 682 | 416 | 679 | 1498 | 700 | 882 | 639 | 805 | 939 |
| Antiparkinsonian medications | Levodopa  Entacapone  Amantadine Zonisamide | Levodopa  Entacapone  Amantadine | Levodopa  Ropinirole  Rotigotine | Levodopa Cabergoline  Entacapone Amantadine  Zonisamide | Levodopa  Rasagiline  Zonisamide | Levodopa  Pramipexole  Rasagiline  Entacapone | Levodopa  Rotigotine  Rasagiline | Levodopa  Rotigotine  Selegiline  Zonisamide | Levodopa  Rotigotine  Rasagiline |
| Sleep-related medications | Clonazepam |  |  |  | Suvorexant |  |  |  |  |

No changes in antiparkinsonian or sleep-related medications occurred during the 12-week treatment period.

LEDD, levodopa equivalent daily dose; MMSE, Mini-Mental State Examination; MoCA-J, Japanese version of the Montreal Cognitive Assessment.

**Supplementary Table S2. PDSS-2 subdomain scores at baseline and week 12.**

| **PDSS-2 domain** | **Baseline** | **Week 12** | **p value** | **Effect size (r)** |
| --- | --- | --- | --- | --- |
| Motor symptoms at night | 11 [8–16] | 8 [6–13] | 0.092 | 0.56 |
| PD-related symptoms at night | 6 [2–12] | 3 [0–8] | 0.029 | 0.73 |
| Disturbed sleep | 12 [10–14] | 6 [5–12] | 0.028 | 0.73 |

Data are presented as median [interquartile range].

PD, Parkinson’s Disease; PDSS-2, Parkinson’s Disease Sleep Scale-2.

**Supplementary Table S3. PDQ-39 subdomain index at baseline and week 12.**

| **PDQ-39 domain** | **Baseline** | **Week 12** | **p value** | **Effect size (r)** |
| --- | --- | --- | --- | --- |
| Mobility | 47.5 [25.0–55.0] | 30.0 [25.0–47.5] | 0.553 | 0.20 |
| Activities of daily living | 33.3 [16.7–70.8] | 25.0 [12.5–33.3] | 0.058 | 0.63 |
| Emotional well-being | 41.7 [29.2–58.3] | 33.3 [12.5–41.7] | 0.030 | 0.72 |
| Stigma | 25.0 [12.5–43.8] | 6.2 [0.0–25.0] | 0.022 | 0.76 |
| Social support | 8.3 [0.0–33.3] | 8.3 [0.0–25.0] | 0.343 | 0.32 |
| Cognition | 43.8 [37.5–50.0] | 25.0 [12.5–50.0] | 0.068 | 0.61 |
| Communication | 33.3 [8.3–66.7] | 8.3 [0.0–33.3] | 0.035 | 0.70 |
| Bodily discomfort | 41.7 [33.3–58.3] | 33.3 [0.0–41.7] | 0.036 | 0.70 |

Data are presented as median [interquartile range].

PDQ-39, Parkinson’s Disease Questionnaire-39.

**Supplementary Table S4. Additional polysomnographic parameters at baseline and week 12.**

| **Parameter** | **Baseline** | **Week 12** | **p value** | **Effect size (r)** |
| --- | --- | --- | --- | --- |
| Total recording time (min) | 556.0 [515.5–594.5] | 560.5 [549.5–598.0] | 0.722 | 0.12 |
| REM latency (min) | 158.5 [0.0–274.0] | 267.0 [140.5–322.5] | 0.407 | 0.28 |
| Arousal index (/h) | 14.7 [10.4–20.4] | 18.3 [17.5–26.3] | 0.155 | 0.47 |
| Apnea–hypopnea index (/h) | 7.5 [3.4–12.4] | 6.5 [3.9–17.2] | 0.722 | 0.12 |
| Oxygen desaturation index (/h) | 1.6 [1.1–11.9] | 1.9 [1.1–7.7] | 0.678 | 0.14 |
| PLMS index (/h) | 0.8 [0.0–3.7] | 1.2 [0.0–16.8] | 0.397 | 0.00 |
| PLMS arousal index (/h) | 0.0 [0.0–0.2] | 0.4 [0.0–0.5] | 0.675 | 0.00 |

Data are presented as median [interquartile range].

PLMS, periodic limb movements in sleep; REM, rapid eye movement.
